# Mitochondrial haplogroup R offers protection against obesity in Kuwaiti and Qatari populations

**DOI:** 10.3389/fendo.2024.1449374

**Published:** 2024-10-11

**Authors:** Mohammed Dashti, Naser M. Ali, Hussain Alsaleh, Sumi Elsa John, Rasheeba Nizam, Fahd Al-Mulla, Thangavel Alphonse Thanaraj

**Affiliations:** ^1^ Genetics and Bioinformatics Department, Dasman Diabetes Institute, Kuwait City, Kuwait; ^2^ Department of Medical Laboratories, Ahmadi Hospital, Kuwait Oil Company (KOC), Ahmadi, Kuwait; ^3^ Saad Al-Abdullah Academy for Security Sciences, Ministry of Interior, Shuwaikh, Kuwait

**Keywords:** obesity, mitochondrial haplogroups, mtDNA mutations, Arabs, Kuwait, Qatar

## Abstract

**Background:**

The Kuwaiti and Qatari populations have a high prevalence of obesity, a major risk factor for various metabolic disorders. Previous studies have independently explored mitochondrial DNA (mtDNA) variations and their association with obesity in these populations. This study aims to investigate the role of mtDNA haplogroups and variants in obesity risk among these Gulf populations.

**Methods:**

Whole exome sequencing data from 1,112 participants (348 Kuwaitis and 764 Qataris) were analyzed for mtDNA variants. Participants were classified as obese or non-obese based on body mass index (BMI). Association analyses were performed to examine the relationship between mtDNA haplogroups and obesity, adjusting for covariates such as age and sex.

**Results:**

Haplogroup R was found to be protective against obesity, with an odds ratio (OR) of 0.69 (p = 0.045). This association remained significant after adjusting for age and sex (OR = 0.694; 95% CI: 0.482-0.997; p = 0.048). Several mtDNA variants, particularly those involved in mitochondrial energy metabolism, showed nominal associations with obesity, but these did not remain significant after correcting for multiple testing.

**Conclusion:**

Haplogroup R consistently demonstrates a protective association against obesity in both Kuwaiti and Qatari populations, highlighting its potential as a biomarker for obesity risk in the Gulf region. However, further research with larger sample sizes is needed to validate these findings and clarify the role of mtDNA variants in obesity.

## Introduction

Mitochondria are membrane-bound organelles that play a critical role in the generation of the metabolic energy in cells through the process of oxidative phosphorylation. Additionally, mitochondria are key components in calcium buffering, heme biosynthesis, iron homeostasis and regulation of cellular apoptosis (1). Hence, the mitochondrial functions play an important role in cellular and metabolic health and homeostasis. Mitochondrial proteins are encoded by both the nuclear DNA and the mitochondrial DNA (mtDNA). mtDNA is a 16.6 kb circular double-stranded DNA that encodes 37 genes that are important for normal mitochondrial function (2).

Altered mitochondrial function has been implicated in multiple disorders, including dementia, epilepsy, strokes, Parkinson disease, ataxia, cardiomyopathy, coronary artery disease, chronic fatigue syndrome, primary biliary cirrhosis, and ovarian dysfunction (3). Moreover, many studies have connected mitochondrial dysfunction with metabolic diseases, including type 2 diabetes and obesity (4–7). Obesity is a chronic inflammatory disease that recognized as a major public health concern. The pivotal role of mitochondria in metabolic signaling processes, such as ATP levels, oxidative stress, ER stress and inflammation, underscores their influence on obesity (6). *In vitro* experiments conducted by Yin et al. (8) revealed that adipocyte mitochondrial oxidative capacity is reduced in obese compared to lean adults, suggesting that obesity is related to mitochondrial dysfunction (8). A more recent study by Bordoni et al. (9) illustrated changes in mtDNA copy number in overweight/obese group compared to those with normal-weight (9).

Pathogenic changes in either mtDNA or nuclear DNA, following patterns of autosomal dominant, autosomal recessive, X-linked, *de novo*, or maternal inheritance, are linked to mitochondrial disorders (47). Historically, the identification of such changes was pursued using Sanger sequencing. However, with the emergence of advanced high-throughput sequencing technologies, next-generation sequencing (NGS) has become the preferred method for examining mitochondrial variations (10–12). In recent work, we showcased the application of whole exome sequencing (WES) to evaluate entire mitochondrial haplogroups and their potential connection to obesity risk in both Kuwaiti and Qatari populations (7, 13).

Growing evidence in literature suggests association of mitochondrial DNA variants and haplogroups with obesity in various ethnic populations (2, 14–16, 48, 17). In the current study, we aim to investigate the contribution of mitochondrial variants and haplogroups to the risk of obesity in Arabs from the Gulf region which is marked by high prevalence of obesity (18). To this end, we employed WES data from Kuwait and Qatar. These nations are not only geographically and ethnically aligned but also showcase significant overlap in the genetic makeup of their population subgroups (19–23) and in their maternal lineage structures (7, 13).

## Materials and methods

### Ethics statement

The institutional Ethical Review Committee at Dasman Diabetes Institute reviewed and approved this study in accordance with the declaration of Helsinki (2008 amendments). The human whole-exome sequence data used in this study were previously published as separate studies in 7, 13, 24, 25. The original studies obtained written informed consent of participants who were recruited under protocols approved by the Institutional Review Boards of Hamad Medical Corporation and Weill Cornell Medical College in Qatar for Qatari samples.

### Study exome data

The study cohort comprised a total of 1,112 Kuwaiti and Qatari individuals. Whole exomes of the 348 Kuwaiti participants of the cohort were sequenced on Illumina Hiseq platform (Illumina Inc. USA) using Illumina capture kits as described in (25) and available at Sequence Read Archive (accession: SUB12360411). A subset of these samples (288 individuals) was previously published in 13. Whole exomes of the 764 Qatari participants with available clinical characteristic were used in the present research. These samples (**Supplementary Table 1**) were previously sequenced on Illumina Hiseq platform (Illumina Inc. USA) using Agilent capture kits that captured the whole mitochondria genome (24). The combined dataset of 1,112 Kuwaiti and Qatari individuals was divided into obese and non-obese groups based on body mass index (BMI); 667 individuals BMI of ≥ 30 kg/m^2^ were considered obese and 445 with BMI < 30 kg/m^2^ were considered non-obese.

### Derivation of mtDNA sequences, calling variants, annotation and classification of haplogroups

The whole exome sequencing data were aligned to the GRCh37 human genome assembly using the Burrows-Wheeler Aligner (BWA-MEM) with default parameters (26). After removing duplicated reads, mtDNA sequences (NC_012920.1), which correspond to the Revised Cambridge Reference Sequence (rCRS), were extracted using Picard tool version 2.20.2 (http://broadinstitute.github.io/picard) and SAMtools tool version 0.1.19 (27). mtDNA coverage and Genomic Variant Call Format (GVCF) were created for each sample using Genome Analysis Tool Kit (GATK) version v3.8-1-0 (28). GATK haplocaller was used to genotype a combined 287 GVCF files to identify mtDNA variants in Variant Calling Format (VCF) file. By way of using HaploGrep 2 tool (29) with phylotree build 17 (accessed on 16 January 2022), the mitochondrial haplogroup profiling mtDNA VCF files was carried out for each of the 1,112 samples. mtDNA annotation was carried out using Ensembl Variant Effect Predictor (VEP) (49) and Mitomap (30).

### Statistical analyses

In this work, the power calculation was conducted based on the proportions of obese and non-obese individuals within the combined cohort from Kuwait and Qatar. We observed that 59.98% (p1 = 0.5998) of the population were obese, while 40.02% (p2 = 0.4002) were non-obese. The effect size determined for this dataset was 0.4020. Our statistical power analysis, conducted at a significance level of α=0.05, revealed a power of 99.99% (0.9999), indicating a highly reliable probability of detecting the observed variance in obesity rates between classifications.

Descriptive statistics for the demographic and anthropometric data were carried out using R software version 3.6.2 (https://www.R-project.org/). Continuous variables, such as age and BMI scores, were presented as mean ± standard deviation (SD), median, and interquartile (IQ). Chi-square test was used to assess the statistical significance of associations between the categorical parameters (sex and nationality) with obesity. Furthermore, age and BMI scores were used to test their associations with obesity using Mann-Whitney U test.

Principal component analysis (PCA) was implemented to check if there exists any hidden relationship among the samples due to covariates which could eventually lead to spurious results. PCA was conducted using the whole mtDNA variants, and the result was visualized on biplot using principal components (PC) that carry the most variation in the data. The PCA was conducted using *PCAtools* of the R software package.

To test for nominal association between obesity and mtDNA haplogroups, Fisher’s exact test was used. Both the odds ratio (OR) and 95% confidence intervals (C.I.) values for each haplogroup were calculated and *p*-value <0.05 was used as the cut-off for statistical significance. To adjust for covariates (sex, age and nationality) logistic regression was implemented using IBM^®^ SPSS^®^ Statistics Version 25 software. Finally, PLINK version 1.9 (31) was used to find mtDNA variants associated with obesity, and the significant cut-off value used was a two-tailed *p*-value <0.05.

## Results

### Study population

**Table 1** presents descriptive statistics for the dataset containing 1,112 Kuwaiti and Qatari individuals. Mann-Whitney U test displayed no significant difference between obese and non-obese individuals with regards to age and nationality status. However, the Chi-squared test showed a significant result for the distribution of obese and non-obese individuals with regards to gender (*p*-value <0.05). Both cohorts in our study, sourced from prior research (24, 25), underwent relatedness assessments based on nuclear DNA to ensure unrelated samples.

**Table 1:** Demographic and Anthropometric Data pertaining to the study subjects.

	Total (N=1,112) n(%)	Non-obese (N=445) n(%)	Obese (N=667) n(%)	p-value
Gender				
Male	470 (42%)	146 (33%)	324 (49%)	2.568E-07
Female	642 (58%)	299 (67%)	343 (51%)	
Nationality				
Kuwaiti	348 (31)	153 (34.4)	195 (29.2)	8.10E-02
Qatari	764 (69)	292 (66)	472 (70.8)	
Age (years)				
<=50	501 (45%)	206 (46%)	295 (44%)	0.054
>50	611 (55%)	239 (54%)	372 (56%)	
Mean ± SD	52 (12.6)	51 (13.7)	52 (11.7)	
Median (IQ)	52 (43-60)	52 (40-60)	53 (44-61)	
BMI score				
Mean ± SD	32.8 (7.4)	26.1 (2.8)	37.2 (6)	<2E-16
Median (IQ)	32 (28-37)	27 (24-29)	36 (32-41)	

p-value for age categories and BMI scores for case vs control were calculated using Mann–Whitney U test. p-values for differences in sex distribution in case vs control groups were calculated using Chi-squared test.

### Mitochondrial haplogroup association with obesity

On average, the mitochondrial DNA sequence coverage for 1,112 whole exome samples was 75X. In addition, the number of mtDNA variants (SNPs and INDELs) identified was 1,850. A set of 15 mitochondrial haplogroups were identified (H, HV, J, K, L, M, N, R, T, U, W, X, B, E, and I) using HaploGrep 2 tool with average haplogroup quality scores of 93% (**Table 2**).

**Table 2:** Mitochondrial haplogroups association for obesity in Kuwaiti and Qatari populations.

Haplogroup	Kuwait	Qatar	Total	Obese	Non-obese	OR	p-value	OR (95%CI)* after adjusting the model for age and sex	p-value* after adjusting the model for age and sex
	N (348)	N (764)	N (1,112)	N (667)	N (445)				
H	49 (14.08%)	79 (10.34%)	128 (11.51%)	70 (10.49%)	58 (13.03%)	0.78	0.194	0.796 (0.546-1.161)	0.236
HV	14 (4.02%)	40 (5.04%)	54 (4.86%)	30 (4.5%)	24 (5.39%)	0.83	0.496	0.812 (0.465-1.420)	0.466
J	62 (17.82%)	109 (14.27%)	171 (15.38%)	111 (16.64%)	60 (13.48%)	1.28	0.153	1.283 (0.909-1.811)	0.157
L	49 (%14.08)	89 (11.65%)	138 (12.41%)	88 (13.19%)	50 (11.24%)	1.2	0.332	1.178 (0.809-1.715)	0.393
M	18 (5.17%)	57 (7.46%)	75 (6.74%)	44 (6.6%)	31 (6.97%)	0.94	0.810	0.907 (0.560-1.470)	0.693
N	23 (6.61%)	30 (3.93%)	53 (4.77%)	31 (4.65%)	22 (4.94%)	0.94	0.820	0.884 (0.501-1.561)	0.671
R	42 (12%)	96 (12.57%)	138 (12.41%)	72 (10.79%)	66 (14.83%)	0.69	0.045	0.694 (0.482-0.997)	0.048
T	24 (6.90%)	83 (10.86%)	107 (9.62%)	65 (9.75%)	42 (9.44%)	1.04	0.865	1.049 (0.694-1.587)	0.819
U	40 (11.49%)	107 (14.01%)	147 (13.22%)	96 (14.39%)	51 (11.46%)	1.3	0.157	1.308 (0.904-1.891)	0.154
X	12 (3.45%)	27 (3.53%)	39 (3.51%)	18 (2.7%)	21 (4.72%)	0.56	0.073	0.584 (0.305-1.119)	0.105
Others (B,E,W,K,I)	15 (4.3%)	47 (6.15%)	62 (5.58%)	42 (6.3%)	20 (4.49%)	1.43	0.199	1.554 (0.890-2.715)	0.121

*Values after adjustment for age and sex. N, number of individuals; OR, odds ratio; CI, confidence intervals for OR as calculated using logistic regression model using PLINK.

**Figure 1:**
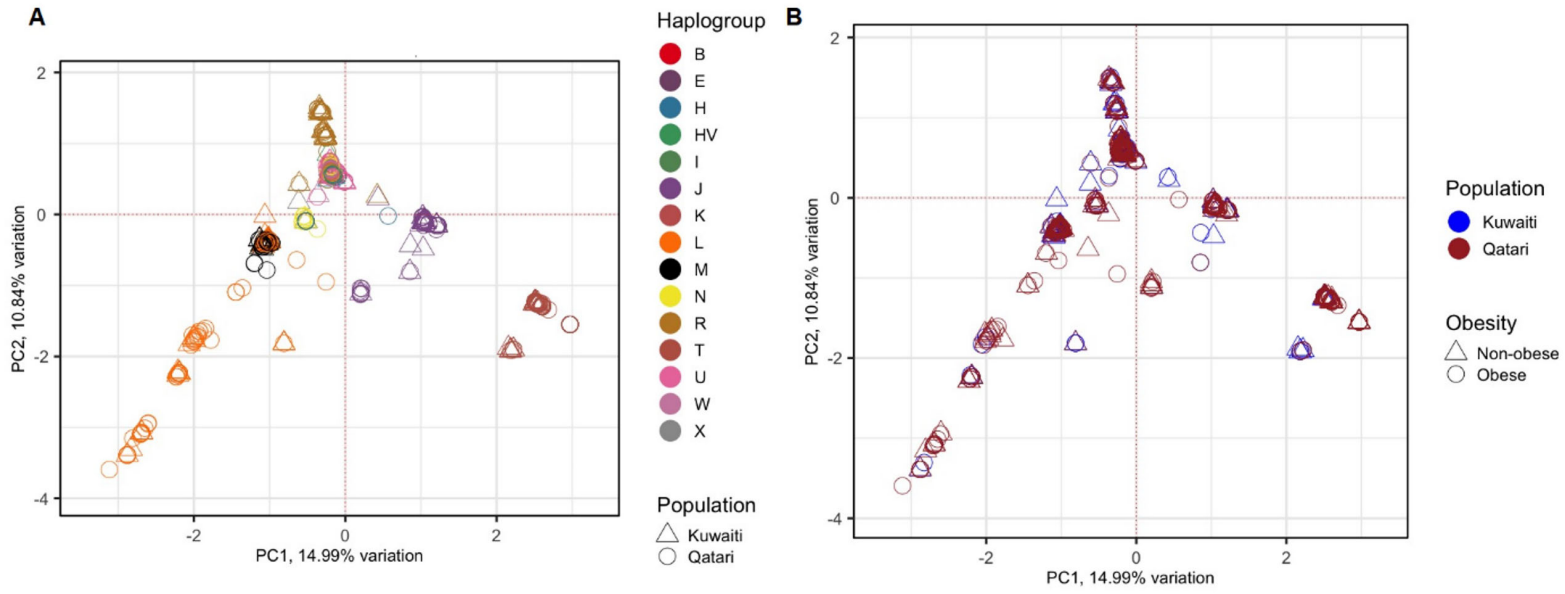
PCA of 1,112 Kuwaiti and Qatari samples based on their mtDNA variants. Different colours and shapes of the samples indicate different groups as shown in both plots **(A, B)**. In both the plots, the principal components 1 (PC1) and 2 (PC2) were the first two axes of PCA and represented 14.99% and 10.84% variance, respectively. In **(A)**, the samples were coloured based on their mitochondrial haplogroups and their ancestries. In **(B)**, the samples were coloured based on their population ancestries and obesity status.

To avoid spurious results due to the presence of hidden relationships between the samples, distribution of the samples was tested using PCA based on 1,850 mitochondrial variants. The 1,112 samples clustered based on their maternal haplogroups (**Figure 1A**), which indicates how mtDNA is rich in ancestral information. However, as shown in **Figure 1B**, the samples did not cluster based on their ancestries (Kuwaiti or Qatari), or by obesity status.

**Figure 2:**
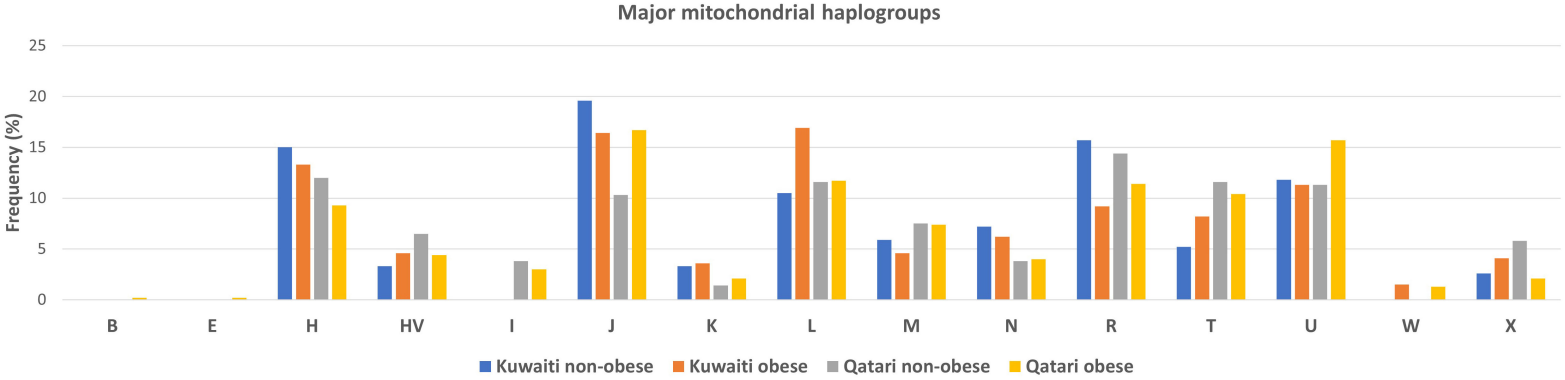
Frequency distribution of major mtDNA haplogroups in obese and non-obese groups for Kuwaiti and Qatari populations. The figure displays the distribution of major mtDNA haplogroups across obese and non-obese groups separately for Kuwaiti and Qatari populations, illustrating the variation in haplogroup frequencies, particularly haplogroup R, which shows a higher prevalence in non-obese groups in both populations.

Frequencies of the mitochondrial haplogroups as seen in the combined and individual groups of Kuwaiti and Qatari subjects are presented in **Table 2**. Minor haplogroups with frequency less than 3% were grouped as “others”. The most common mitochondrial haplogroups seen within this investigation was J haplogroup, followed by U, L, R haplogroups. Results of mitochondrial haplogroup association tests (**Table 2**) show that individuals with R haplogroup had protective effect against developing obesity (OR = 0.69; *p* = 0.045). In addition, after adjusting for age and sex for individuals with R maternal haplogroup using multivariate logistic regression, the *p*-value remained significant (OR/95% C.I = 0.694/0.482-0.997; *p* = 0.048). The frequency of R haplogroup in Qatari cohort is 12.6% with the Qatari subgroups of obese and non-obese individuals having frequency values of 11.4% and 14.4%, respectively. In a similar manner, the frequency of R haplogroup in Kuwaiti cohort is 12% with the Kuwaiti subgroups of obese and non-obese individuals having frequency values of 9.2% and 15.7%, respectively. **Figure 2** shows the frequency distribution of major mtDNA haplogroups across obese and non-obese groups in both Kuwaiti and Qatari populations. The R and H haplogroups are more prevalent in the non-obese groups compared to the obese groups in both populations. Moreover, no other haplogroups showed significant association trends that were consistent across both populations. Most of the individuals who carried the R haplogroup in our study belonged to the R0a subclade (**Table 3**).

**Table 3:** Sub-clade distribution of individuals carrying mitochondrial haplogroup R.

Sub-clade	Number of individuals
R	1
R0a1	44
R0a2	66
R0a3	1
R2 + 1	6
R2a	5
R30	1
R30a	2
R30b	2
R5a2	1
R6a2	1
R8a1	4
R8a2	2
R8b1	2
Total	138

### Mitochondrial variant association with obesity

Multiple logistic regression test was carried out to identify the variants associating with obesity at *p*-values < 0.05 (**Table 4**). We observed a set of 37 mtDNA variants to have nominal associations (*p*-value ≤ 0.05) with obesity, with models adjusted for age, sex, mitochondrial haplogroup and nationality. **Table 4** shows that 17 out of 37 mtDNA variants were positively correlated with the risk of obesity, and 20 were negatively correlated with risk of obesity. All the variants exhibited variable frequencies across obese, and non-obese groups as well as R haplogroups. Conditional analysis using the top leading variants with the lowest *p*-value from (MT:16304T>C) (**Table 4**), identified 23 of the 37 variants remained significant. This suggests that these 23 variants (**Table 4** footnote) have an independent effect on the phenotype with respect to the conditioned SNP. Therefore, we assume that the remaining 14 mitochondrial variants from **Table 4**, which lost their significance, are dependent on the top mitochondrial variant.

**Table 4:** Mitochondrial DNA variants associated with obesity in Arabs from Kuwait and Qatar.

mtDNA variants^@^	Gene	Consequence	Frequency in Obese group	Frequency in Non-obese group	Frequency in R haplogroup	OR (95% CI)*	p-value
MT:16304T>C	TP	Upstream	0.02	0.05	0.18	0.389 (0.198-0.762)	0.006
○*MT:750G>A*	RNR1	Non-coding	0.02	0.04	1	0.389 (0.181-0.837)	0.016
○*MT:15218A>G*	CYB	Missense	0.01	0.03	0	0.319 (0.124-0.82)	0.018
*•MT:5460G>A*	ND2	Missense	0.08	0.04	0.01	1.945 (1.121-3.373)	0.018
*MT:15326G>A*	CYB	Missense	0	0.02	0.95	0.152 (0.031-0.752)	0.021
*MT:14040G>A*	ND5	Synonymous	0.01	0.03	0.007	0.316 (0.115-0.869)	0.026
*MT:7673A>G*	CO2	Missense	0.01	0.03	0	0.349 (0.138-0.883)	0.026
*MT:10142C>T*	ND3	Synonymous	0.01	0.03	0	0.344 (0.132-0.896)	0.029
*MT:5360C>T*	ND2	Synonymous	0.01	0.03	0	0.345 (0.133-0.896)	0.029
*MT:8137C>T*	CO2	Synonymous	0.01	0.03	0	0.345 (0.133-0.898)	0.029
*MT:3741C>T*	ND1	Synonymous	0.01	0.03	0	0.347 (0.133-0.903)	0.030
*MT:8684C>T*	ATP6	Missense	0.01	0.03	0	0.347 (0.134-0.904)	0.030
○*MT:4216T>C*	ND1	Missense	0.29	0.23	0.08	1.370 (1.028-1.825)	0.032
*MT:14110T>C*	ND5	Missense	0.01	0.03	0	0.334 (0.123-0.91)	0.032
•MT:11719A>G	ND4	Synonymous	0.24	0.3	0.2	0.717 (0.529-0.972)	0.032
○*MT:5501A>G*	ND2	Synonymous	0.02	0	0	5.050 (1.136-22.45)	0.033
○*MT:153A>G*	RNR1	Upstream	0.02	0.05	0	0.469 (0.23-0.957)	0.037
○MT:11914G>A	ND4	Synonymous	0.11	0.07	0.01	1.590 (1.026-2.466)	0.038
*MT:12358A>G*	ND5	Missense	0.03	0.01	0	3.211 (1.064-9.694)	0.039
*MT:14769A>G*	CYB	Missense	0.01	0.02	0	0.292 (0.09-0.945)	0.040
MT:15907A>G	TT	Non-coding	0.03	0.01	0	3.165 (1.045-9.585)	0.042
MT:6045C>T	CO1	Synonymous	0.03	0.01	0	3.155 (1.042-9.558)	0.042
MT:5390A>G	ND2	Synonymous	0.03	0.01	0	3.152 (1.041-9.546)	0.042
MT:10876A>G	ND4	Synonymous	0.03	0.01	0	3.152 (1.041-9.546)	0.042
MT:13734T>C	ND5	Synonymous	0.03	0.01	0	3.152 (1.041-9.546)	0.042
MT:3720A>G	ND1	Synonymous	0.03	0.01	0	3.151 (1.041-9.54)	0.042
*MT:6915G>A*	CO1	Missense	0.01	0.03	0	0.376 (0.146-0.967)	0.042
*MT:194C>T*	TF	Upstream	0.02	0	0	8.264 (1.069-63.89)	0.043
•MT:16362T>C	TP	Upstream	0.15	0.21	0.8	0.713 (0.513-0.99)	0.043
○MT:16051A>G	TP	Upstream	0.11	0.07	0	1.617 (1.014-2.58)	0.044
*MT:93A>G*	TF	Upstream	0.05	0.02	0.003	2.196 (1.022-4.719)	0.044
*MT:8664A>G*	ATP6	Synonymous	0.01	0.03	0	0.379 (0.147-0.973)	0.044
MT:217T>C	TF	Upstream	0.03	0.01	0	3.123 (1.031-9.464)	0.044
○*MT:13966A>G*	ND5	Missense	0.03	0.05	0	0.504 (0.259-0.983)	0.045
MT:6152T>C	CO1	Synonymous	0.03	0.01	0	2.765 (1.011-7.563)	0.048
MT:10685G>A	ND4L	Synonymous	0.02	0	0.08	7.863 (1.02-60.6)	0.048
○*MT:14470T>C*	ND6	Synonymous	0.03	0.05	0	0.501 (0.252-0.996)	0.049

^@^mtDNA variants in italics were identified through conditional analysis as having an independent effect on obesity with respect to the conditioned SNP (MT:16304T>C) with the lowest p-value.

^*^OR, odds ratio; CI, confidence intervals, •, Variant reported in the Kuwaiti study (13), ○, Variant reported in the Qatari study (7).

Furthermore, we identified DNA mitochondrial variants that were present only in obese individuals (in at least 7 individuals) but were not found in the non-obese individuals (**Table 5**). The identified mtDNA variants were checked against Mitomap and ClinVar databases for potential pathogenic significance relating to obesity, BMI, and body fat and were found to have no such pathogenic impact.

**Table 5:** Mitochondrial variants associated with obesity of Arabs in Kuwaiti and Qatari populations.

SNP	Gene	Consequence	Frequency in obese group	Number of individuals
MT:8994G>A	ATP6	Synonymous	0.02	12 individuals
MT:1243T>C	RNR1	Non-coding	0.01	11 individuals
MT:11947A>G	ND4	Synonymous	0.01	10 individuals
MT:16288T>C	TP	Upstream	0.01	30 individuals
MT:15262T>C	CYB	Synonymous	0.01	7 individuals

## Discussion

In our previous studies, we identified associations between specific mitochondrial haplogroups and obesity in Kuwaiti (13) and Qatari (7) populations. These studies demonstrated significant associations with obesity for different maternal haplogroups in these populations within the Arabian Gulf region. The observed variations in obesity-associated haplogroups could be due to several factors, with sample size being a primary consideration. By combining the datasets from Kuwaiti and Qatari populations, we aimed to increase the reliability of our findings and determine whether observed associations are consistent across the broader Gulf region.

An innovative aspect of our study lies in the integration of data from the Kuwaiti and Qatari cohorts. Although these are two distinct countries with their unique characteristics, they share substantial similarities in genetic and environmental factors. Combining data from these populations provides a more comprehensive analysis, enhancing statistical power and offering a broader view of the genetic landscape in the Gulf region. This approach is essential for identifying shared genetic markers and risk factors that may not be evident when analyzing each population in isolation, thereby providing a deeper understanding of obesity risk factors specific to this region.

In this study, samples from both Kuwaiti and Qatari cohorts showed a distinct clustering pattern in the PCA analysis of mitochondrial haplogroups (**Figure 1**). This pattern aligns with our earlier PCA analysis on DNA variants using whole exome data (23). These findings suggest that the mitochondrial DNA of Kuwaiti and Qatari populations share sufficient ethnic homogeneity, allowing us to combine the two cohorts and represent them as a unified group for Arabs in the Gulf region.

Our findings indicate that the mitochondrial haplogroup R, representing 12.41% of the total samples, is significantly more prevalent in the non-obese cohort than in the obese group (**Table 2**, **Figure 2**). The subclade R0a is observed in 80% of individuals with the maternal haplogroup R (**Table 3**). The frequencies of haplogroup R and subclade R0a in our study align with values previously reported in the Arabian Peninsula (32, 33). When we compared our findings on the mitochondrial haplogroup R’s association with obesity against prior studies (14, 34–36), it became evident that this significant association is distinct to Arabs in the Gulf region. We had earlier shown that haplogroup R offers a protective effect against obesity among Kuwaiti individuals (13). A pivotal mutation for the R0a clade is MT:16362T > C (37). This mutation is more prevalent in the non-obese group and is linked to a protective effect against obesity, as determined by multivariate logistic regression analyses of mitochondrial DNA variants, adjusted for age, sex, maternal haplogroup, and nationality. Furthermore, many variants with a strong negative correlation to obesity are infrequent in the R haplogroup. Our study’s most prominent mitochondrial variant was MT:16304T>C, a defining mutation for one of the H sub-haplogroups (38). It’s noteworthy that Haplogroup H was found to significantly reduce the risk of obesity in Kuwait (16). Although haplogroup H is more common in the non-obese group, its significance might be obscured due to variations in study design, such as the BMI criteria used to determine obesity and the sequencing of only the D-loop region (16) rather than the entire genome. Additionally, obesity-associated maternal haplogroups J in Qatar (7) and L in Kuwait (13) were observed at similar frequencies in both obese and non-obese groups in the combined dataset, suggesting that their effects may be influenced by unique genetic, environmental, or lifestyle factors specific to each country. In a combined analysis, these population-specific effects may become diluted, given the broader genetic and environmental landscape. In contrast, haplogroup R, previously identified as protective against obesity in the Kuwaiti study (13), remains significant in our combined analysis, suggesting a consistent effect across Gulf Arab populations. The observed effect size (OR = 0.69) represents a 31% lower risk of obesity, which is meaningful given the complex interplay of genetic and environmental factors in obesity. Even modest protective effects can have substantial public health implications at a population level. However, this association may vary with larger or more diverse samples; thus, further studies are needed to confirm its stability and explore potential modifying factors.

Given its protective association with obesity, haplogroup R has the potential to be developed as a genetic biomarker for assessing obesity risk in the Gulf region. This could support targeted prevention strategies and personalized interventions, such as customized lifestyle modifications, based on an individual’s genetic profile. Further research is needed to explore the clinical utility of haplogroup R in personalized obesity management.

Significant differences in mitochondrial DNA variants between obese and non-obese groups predominantly reside within the complex I nicotinamide adenine dinucleotide (NADH) dehydrogenase subunit genes (MT-ND1, MT-ND2, MT-ND3, MT-ND4, MT-ND4L, MT-ND5, and MT-ND6). NADH dehydrogenase plays a critical role in cell energy production. Thus, variants in its encoding genes, especially MT-ND4:11947A>G found exclusively in obese individuals (**Table 5**), might sway cell energy metabolism, impacting body fat mass, BMI, and obesity risk (34, 39). The oxidative phosphorylation (OXPHOS) system, crucial for cell energy and survival, is influenced by the mitochondrial encoded cytochrome c oxidase I, II (MT-CO1 and MT-CO2) genes, previously linked to obesity (40, 41). In our study, several variants associated with obesity were identified within MT-CO1 and MT-CO2. In the mitochondrial ATP synthase membrane subunit 6 (MT-ATP6) gene, essential for OXPHOS, two exonic variants (MT:8684C>T and MT:8664A>G) were found to associate positively with obesity risk, and one exonic variant (MT:8994G>A) was unique to the obese group. Additionally, two variants (MT:750G>A and MT:153A>G) were found to positively correlate with obesity risk, and one variant (MT:1243T>C) was exclusively found in the obese group within the MT-RNR1 gene, which encodes the MOTS-C protein known for promoting metabolic homeostasis and obesity reduction (42). Lastly, three significant exonic variants and one unique to the obese group were identified in the cytochrome b (MT-CYB) gene; aberrations in this gene have been associated with exercise intolerance (43).

Most of the mitochondrial variants associated with obesity in our study are non-coding and synonymous variants. This could be explained through an epistasis mechanism. Specifically, the combined influences of mitochondrial DNA polymorphisms and nuclear DNA genetic variants might jointly affect overall metabolism, fat storage, diet, and physical health (44, 45).

Our combined analysis identified several mitochondrial DNA variants that remained significant in both the current study and previous studies on Kuwaiti and Qatari populations, including nine variants from Qatar (7) and three from Kuwait (13) with consistent directions of association with obesity. This consistency suggests these variants may play a stable role in influencing obesity risk across Gulf Arab populations. It could also be partially attributed to the changes in sample composition between studies, where the Kuwaiti sample size increased and the Qatari sample size decreased, potentially stabilizing previously observed associations. Additionally, new significant variants found only in the combined analysis may reflect the enhanced statistical power from a larger, integrated dataset, allowing for the detection of both common and population-specific genetic effects on obesity.

The current study has several limitations. First, although the associations between mitochondrial haplogroups (such as the protective effect of haplogroup R) and mitochondrial DNA variants with obesity remained significant after adjusting for covariates, none of these associations passed the Bonferroni correction for multiple testing. This raises the possibility of Type I errors, suggesting that the observed associations, while potentially indicative of genetic influences on obesity, should be interpreted with caution. The p-value for haplogroup R (p = 0.048) is close to the conventional threshold of 0.05, underscoring the need for careful interpretation as results near this threshold can be highly sensitive to variations in study design, sample size, and confounding factors. Therefore, although our findings replicate the protective effect of haplogroup R previously reported in the Kuwaiti cohort (13), further validation in larger, independent cohorts is needed to confirm these results.

Another significant limitation is the lack of comprehensive clinical data to adjust for various health-related confounders. Obesity is a complex disorder influenced by genetic, environmental, and lifestyle factors. The absence of data on lifestyle factors, such as dietary habits, physical activity, and socioeconomic status, may confound the observed genetic associations. For instance, dietary patterns and physical activity levels can significantly affect obesity risk and vary between populations, potentially influencing the observed effects of specific haplogroups. Socioeconomic status, which impacts access to healthy foods, healthcare, and health-related education, is also a crucial determinant of obesity. Including such data would allow for more nuanced insights into the genetic contributions to obesity.

Furthermore, there is no optimized statistical tool available to address the burden of multiple testing in the analysis of mitochondrial variants for complex disorders, although tools exist for rare variant association analysis in exome data (46). While our study’s sample size is comparable to those in previous studies (14, 34–36), increasing the sample size remains essential. A larger sample would help validate the protective effect of haplogroup R, increase the power to detect true associations, and reduce the risk of Type I errors associated with multiple testing. Future research should include a broader range of Arab populations to enhance the robustness and generalizability of these findings.

We aimed to ensure the data’s ethnic and geographical homogeneity to reduce the risk of population stratification. However, potential stratification issues may still exist, as evidenced by the exclusion of minor haplogroups and the need for adjustments for ethnicity. Finally, our findings have not been validated in a larger, independent cohort from the Arabian Gulf, which would provide additional insight into the genetic basis of obesity in this population. Future studies should focus on expanding the cohort size and including more diverse samples to validate these associations and explore their broader applicability.

In conclusion, we have undertaken one of the most comprehensive studies examining the association between mitochondrial haplogroups and variants with obesity in the Kuwaiti and Qatari populations, representing Arabs in the Gulf region. Our findings suggest that individuals carrying the maternal R haplogroup in this region have a decreased risk of developing obesity. Notably, while previous studies have reported associations of obesity with the maternal haplogroup J in Qataris and L haplogroup in Kuwaitis, our combined dataset did not replicate these associations despite the frequencies of these haplogroups being consistent across populations. Moreover, we identified several mitochondrial variants within genes crucial for cellular energy production that show either a protective or risk-enhancing relationship with obesity. This would not only confirm the associations observed in our study but also allow for a more nuanced understanding through sub-haplogroup analyses, potentially unveiling specific genetic factors contributing to obesity in this region.

## Data Availability

The datasets presented in this study can be found in online repositories. The names of the repository/repositories and accession number(s) can be found in the article/**Supplementary Material**.
